# Comparison of long noncoding RNA between muscles and adipose tissues in *Hanwoo* beef cattle

**DOI:** 10.1080/19768354.2018.1512522

**Published:** 2018-12-20

**Authors:** Jae-Young Choi, Donghyun Shin, Hyun-Jeong Lee, Jae-Don Oh

**Affiliations:** a The Animal Molecular Genetics and Breeding Center, Chonbuk National University, Jeonju, Republic of Korea; b Department of Animal Biotechnology, College of Agricultural and Life Sciences, Chonbuk National University, Jeonju, Republic of Korea; c Animal Nutritional & Physiology Team, National Institute of Animal Science, Wanju, Republic of Korea

**Keywords:** Hanwoo, beef cattle, LncRNA, muscle, adipose tissues

## Abstract

Long noncoding RNAs (lncRNAs) regulate the expression of mRNA and can affect various biological processes and phenotypes. Currently, studies of lncRNAs in cattle are under way, but their exact function for several tissues has not yet been established. *Hanwoo* cattle (*Bos taurus coreanae*) have inhabited the Korean peninsula for about 6000 years and are one of the representative domesticated animals in Korea. As a result of intensive breeding, the meat of *Hanwoo* cattle is high in marbling content and is preferred by Koreans and other East Asian people. In this study, the expression of lncRNAs was identified in 36 samples from skeletal muscle and three adipose tissues (intramuscular, subcutaneous, and omental) of nine *Hanwoo* individuals. We identified 76 tissue-specific lncRNAs for each of the four tissues using the differences in expression levels. Through QTL information, we could identify 12 lncRNAs associated with shear force and six lncRNAs associated with body weight, which are two important traits in the *Hanwoo* population breeding strategy. By the physical position comparison of lncRNA and Bovine transcripts information, we could identify 11 lncRNAs that were in bovine transcripts, and four of the 11 genes related to transcripts of lncRNAs were biologically associated with muscle function. We believe this *Hanwoo* lncRNAs study will help reveal the lncRNA role in the physiological mechanisms of these four tissues.

## Introduction

Korean native *Hanwoo* cattle (*Bos taurus coreanae*) are a hybrid of *Bos Taurus* and *Bos zebu*. *Hanwoo* cattle migrated into and settled on the Korean peninsula in about 4000 BC (Han 1996). In the late 1970s, the Korean government launched a *Hanwoo* gene-breeding program to improve the quantity and quality of meat (Lee et al. 2013). Tenderness, taste, and aroma are usually considered to be important criteria for consumers’ purchase of beef (Savell et al. 1987). *Hanwoo* beef is famous for its high marbling content and relatively thin muscle fibers (Kim et al. 1994). Korean consumers are accustomed to the taste of *Hanwoo* beef, with a high content of oleic acid, and prefer it because of the high intramuscular fat content (Kim et al. 1999; Jung and Choi 2003). Thus, *Hanwoo* beef is regarded as the most expensive and delicious beef in Korea (Kim and Lee 2003) and is an important subject of study by many researchers. *Hanwoo* cattle are known to be prolific, but are not good at producing meat and milk because of their slow growth rate and milking capacity. Thus, the Korean cattle industry has aimed to improve their meat-production ability and to increase the number of cattle in order to meet the demand of the growing beef market in Korea (Kim and Lee 2000). In recent years, research has been conducted on muscle fibers and fatty tissues that affect the quality of beef in order to improve the meat quality of *Hanwoo* cattle (Moon et al. 2006; Hwang et al. 2010).

The bovine genome was one of the first decoded mammalian genomes. Because cattle are important farm animals as a major source of nutrients for humans (Tellam et al. 2009). RNA-seq technology was used to identify transcript expression patterns in bovine muscle and various adipose tissues (He and Liu 2013; Sheng et al. 2014).

Transcripts longer than 200 nucleotides that are not translated into protein are defined as long noncoding RNAs (lncRNAs). In previous studies, an lncRNA was thought to be a transcriptional noise like other ncRNAs (Wang et al. 2004; Struhl 2007). However, several studies have shown that the number of lncRNAs found in eukaryotes is increasing (Ulitsky 2016) in recent years. LncRNA is involved in post-transcriptional gene regulation through processes such as RNA maturation, transport, protein synthesis, and transcriptional gene silencing through chromosome regulation (Bernstein and Allis 2005; Whitehead et al. 2009; Geisler and Coller 2013). LncRNAs have a large effect on biological processes, such as cell differentiation, development, immune response, and tumorigenesis, by regulation of mRNA expression (Consortium et al. 2002; Ota et al. 2004; Wilusz et al. 2009; Fan et al. 2015; Gong et al. 2016).

LncRNAs have been studied in many species, including cattle. In cattle, 449 total lncRNAs are located in 405 intergenic regions. This provides a catalog of bovine lncRNAs for gene expression and confer systematic characterization of genomic features (Huang et al. 2012). Deep-transcriptome sequencing studies have identified many lncRNAs in bovine skin specimens (Weikard et al. 2013). The large intergenic noncoding RNAs (lincRNAs) in cattle have been studied, and 584 skeletal-muscle lincRNAs in nine Limousin bull calves were identified (Billerey et al. 2014). Previous studies looked at the metabolic differences between muscle and intramuscular adipose tissues of *Hanwoo* cattle using the RNA-seq technology and a systems biology approach (Lee et al. 2014). Therefore, we can carry out a *Hanwoo* lncRNA study using RNA seq data and other study results.

In this study, we compared the expression patterns of lncRNA between muscle and adipose tissues of *Hanwoo* cattle to better understand the physiological characteristic in *Hanwoo* meat production. We identified 76 tissue-specific lncRNAs for each of the four types of tissue and found 12 lncRNAs associated with shear force and six lncRNAs associated with the body weight, which are important traits in *Hanwoo* population breeding strategy. Additionally, thorough the physical position comparison of lncRNA and Bovine transcript information, we could identify 11 lncRNAs in Bovine transcripts, and two of these 11 genes related to transcripts of lncRNAs were associated with cow muscle function. This study will clarify the bovine biological characteristics and contribute to the production of high-quality *Hanwoo* cattle. In addition, we want to provide the basis for further research on the molecular biological characteristics in the energy storage and usage of *Hanwoo* cattle.

## Materials and method

### Sample preparation and RNA-seq analysis

All analysis was conducted with data reprocessed from a previous study (Lee et al. 2014). Animal and sample preparation are as follows.: A total of nine (three each of cows, steers, and bulls) *Hanwoo* cattle were used in this study. They were fed the same diet and managed at the same location, the *Hanwoo* Experimental Station in the National Institute of Animal Science, throughout the experiment. The average (± standard deviation) carcass weight of the cattle at slaughter was 430.2 (± 40.66) kg. Immediately after slaughter, muscle, intramuscular adipose tissues, and subcutaneous adipose tissues were separated and sampled. The omental adipose tissue was taken within the lesser curvature of the abomasum. All of the tissue samples were immediately frozen using liquid nitrogen and stored at −80°C until the analysis. Animal use, care, and experimental protocols for this experiment were reviewed and preapproved by the Institutional Animal Care and Use Committee of the National Institute of Animal Science (number 2010-042). Total RNA of tissues was isolated using TRIzol (Invitrogen) and an RNeasy RNA purification kit with DNase treatment (Qiagen). The mRNA was isolated from the total RNA using oligo-dT beads and was reverse transcribed into double-strand cDNA fragments. Constructing and sequencing the RNA-seq library for each sample were carried out based on the protocols of Illumina HiSeq2000 to generate 101 pair-end reads. The quality of RNA-seq reads from all of the tissues was checked using FastQC.

### Analysis of lncRNA discovery

Filtering was conducted to remove the low-quality sequences. The filtered sequences were mapped to a Bovine Taurus genome (bosTau6) using STAR v2.4.0b (Dobin et al. 2013). Expression levels were calculated using Cufflinks v2.2.1. Bovine gene information was used to measure expression levels (Trapnell et al. 2010). The bovine lncRNA analysis was used to conduct mapping with reference to annotated *bos taurus* ensemble ID results (Koufariotis et al. 2015).

### Statistical analysis

Differences in the expression level of each tissue were expressed by Heatmap using R package gplots (v3.0.1) (Warnes et al. 2016). An lncRNA principal component analysis (PCA) plot was used to identify differences between muscle and adipose tissues using Mev (http://mev.tm4.org/) (Howe et al. 2011). The DEGseq R package was used to identify differential expression of lncRNA between muscle and adipose samples (Wang et al. 2009). Significant lncRNAs were identified using cut-off of: log2(fold-change)| ≥ 1 and p-value ≤ 0.001. Tissue-specific differentially expressed lncRNAs statistics analysis used the Prism 5 program (San Diego, CA, USA) (Motulsky 2007). The Venn diagram was used to identify the lncRNA assemblages that were extracted from the four tissues. The Venn diagram was displayed using InteractiVenn (http://www.interactivenn.net/) (Heberle et al. 2015).

### Quantitative trait locus (QTL) analysis

QTL regions for comparative analysis with lncRNAs were identified from the Cattle QTL Database (http://www.animalgenome.org/cgi-bin/QTLdb/BT/index). In the cattle, the QTL associated with the quality and productivity of the meat was selected. The position of the selected QTL was compared with the lncRNA expressed in muscle and adipose tissues.

### Overlapped gene analysis

The location of lncRNA was identified by ensembl biomart (ensembl.org/biomart) using transcript ID. The databases used ensemble Genes 92 Cow genes (UMD3.1). We found for by overlapped gene predicted to be affected by lncRNA. The position information of lncRNA in the genome and the gene position information of the bovine were matched using Python script. Transcription directions of lncRNA and overlapped genes were used as transcript information by ensemble. Identification of pseudogenes was also confirmed through ensemble data base. (ensembl.org).

## Results and discussion

### RNA sequencing information

All analysis was conducted with data reprocessed from a previous study (Lee et al. 2014). RNA-seq raw data information is as follows. Totals of 34.2, 35.8, 35.1, and 38.1 Mb of raw reads were obtained on average from muscle, intramuscular adipose, subcutaneous adipose, and omental adipose tissues, respectively. More than 99.5% of the reads remained after being filtered by the quality control, and more than 95.9% of these were mapped to the reference genome. The average length of lncRNA was 866 bp, the minimum length was 209 bp, and the maximum was 3748 bp.

### Expression patterns of lncRNA in Hanwoo cattle

**Figure 1.**
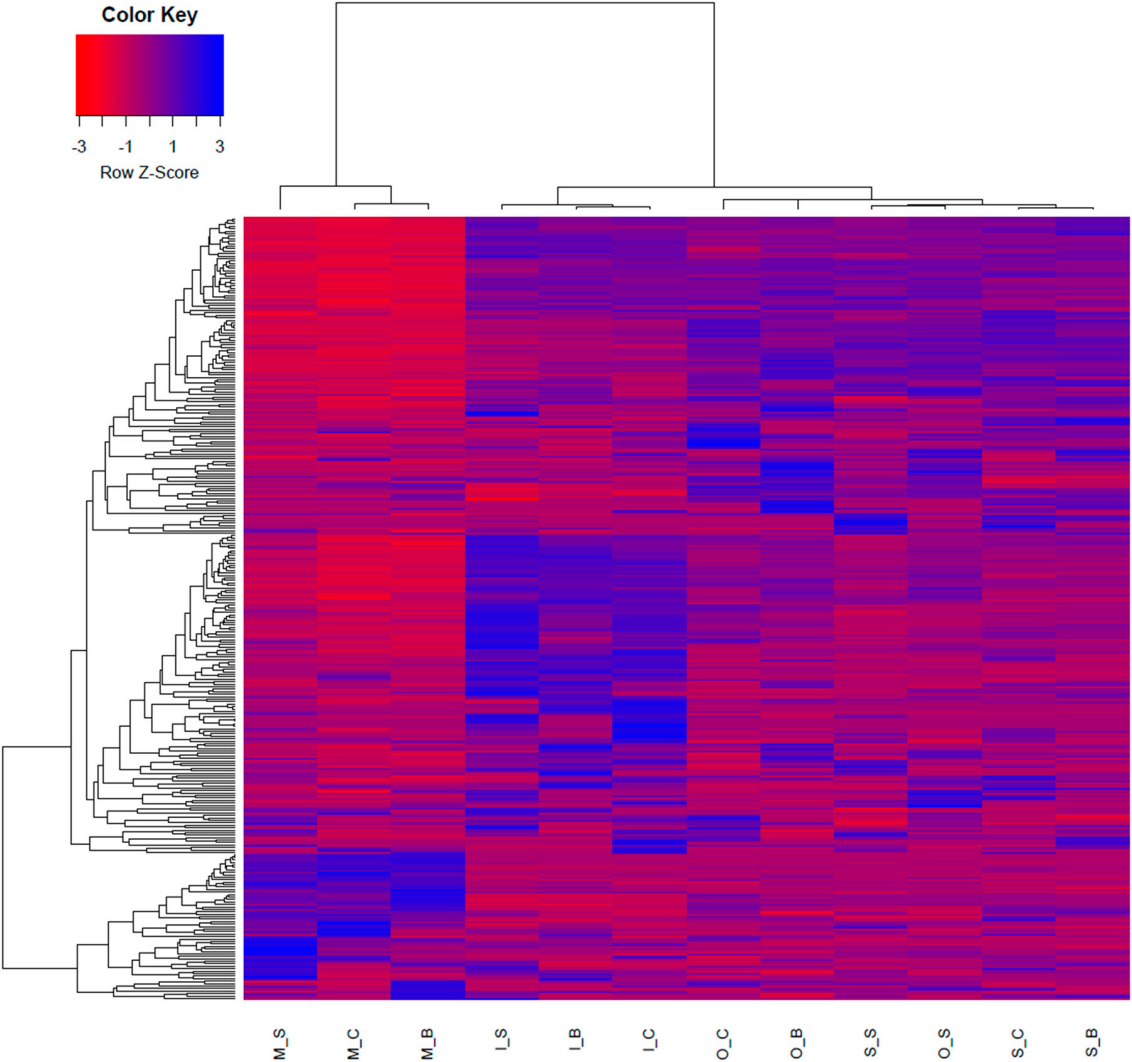
Transcripts expressed by Hanwoo cattle in muscle and adipose tissues. The abbreviations under the bar mean tissues (I, Intramuscular adipose; M, Muscles; S, Subcutaneous adipose; O, Omental adipose) and sex (C, Cow; S, Steer; B, Bull).

**Figure 2.**
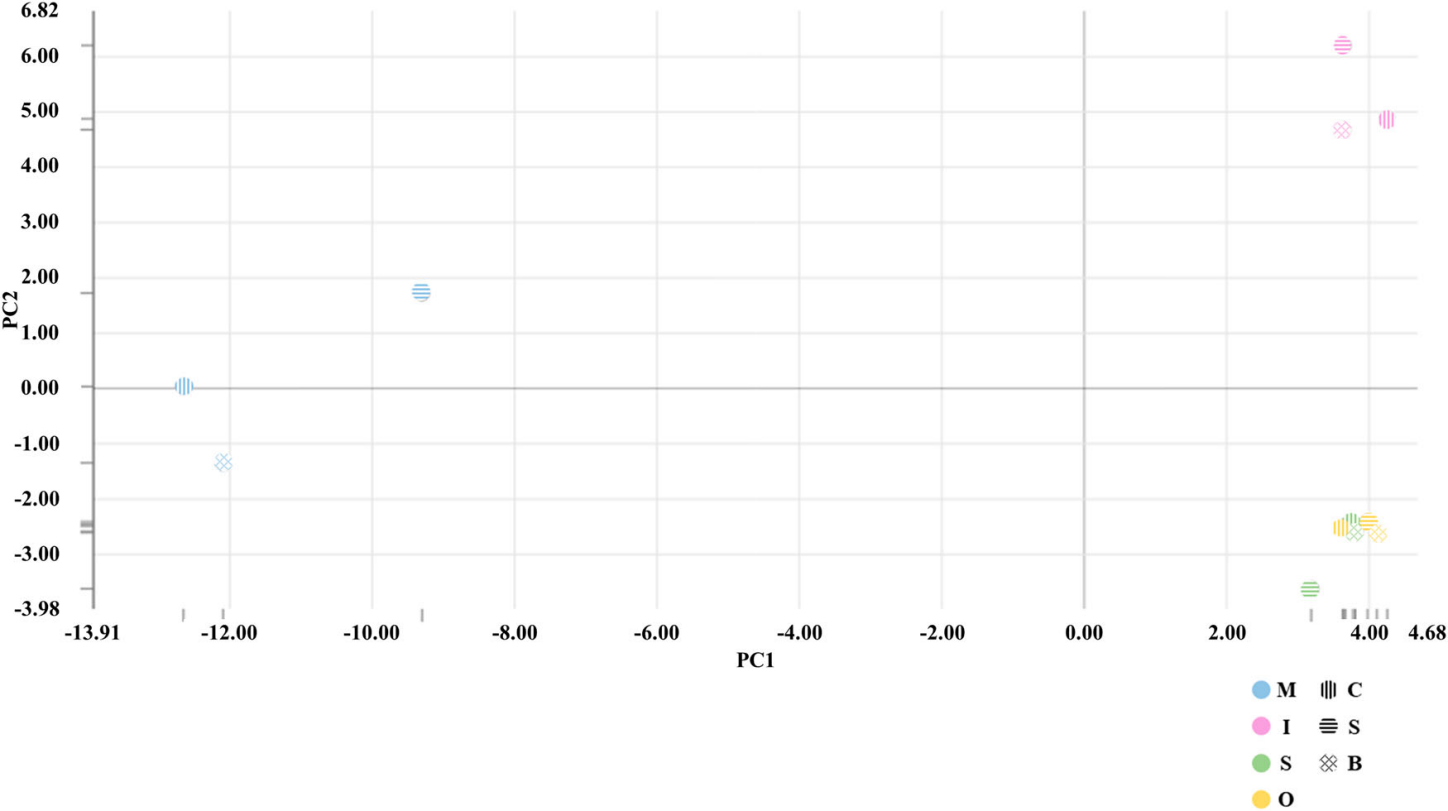
PCA plot of muscle and adipose tissues transcripts. The abbreviations in the colored circles mean tissues (I, Intramuscular adipose; M, Muscles; S, Subcutaneous adipose; O, Omental adipose) and the stripes in the circles mean sex (C, Cow; S, Steer; B, Bull).

**Figure 3.**
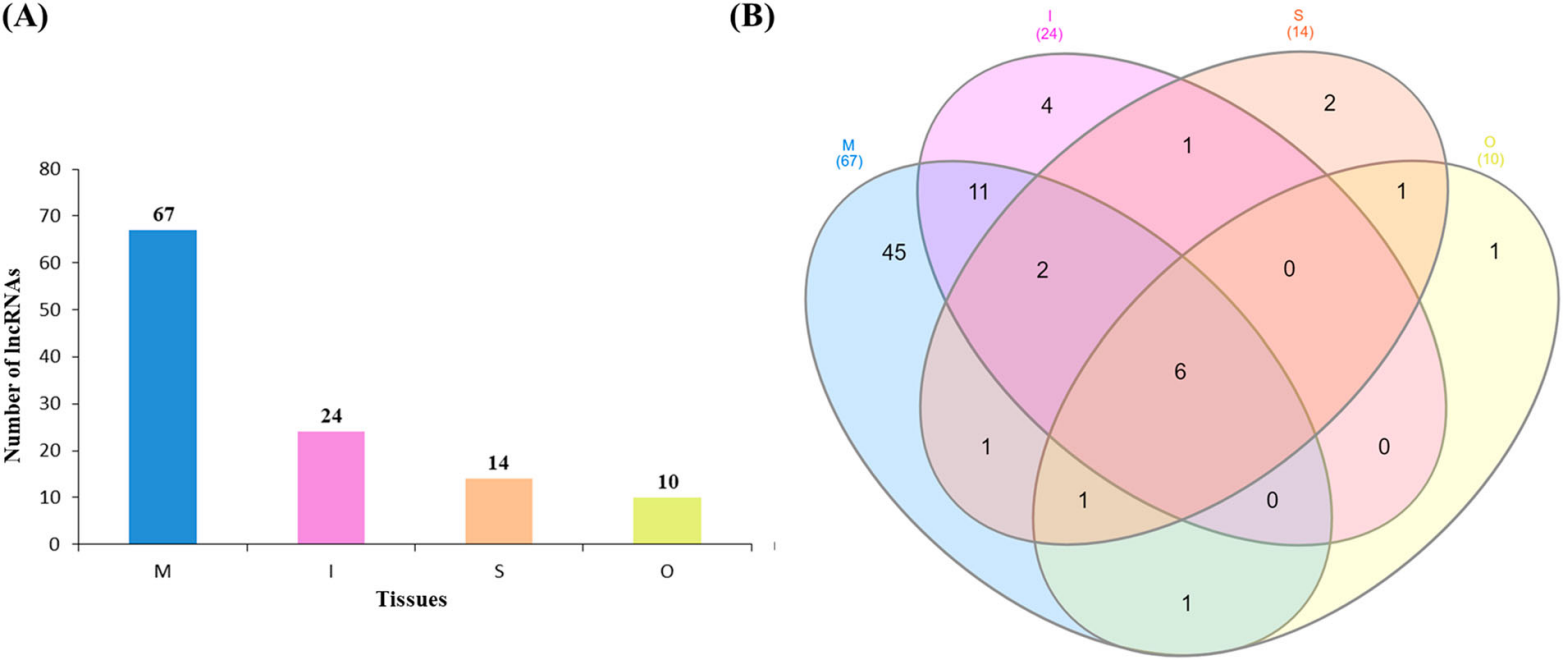
(A) Number of lncRNAs in muscle and adipose tissues. (B) Venn diagram showing the co-expression of lncRNAs muscle and three adipose tissues. The abbreviations mean tissues (I, Intramuscular adipose; M, Muscles; S, Subcutaneous adipose; O, Omental adipose).

**Figure 4.**
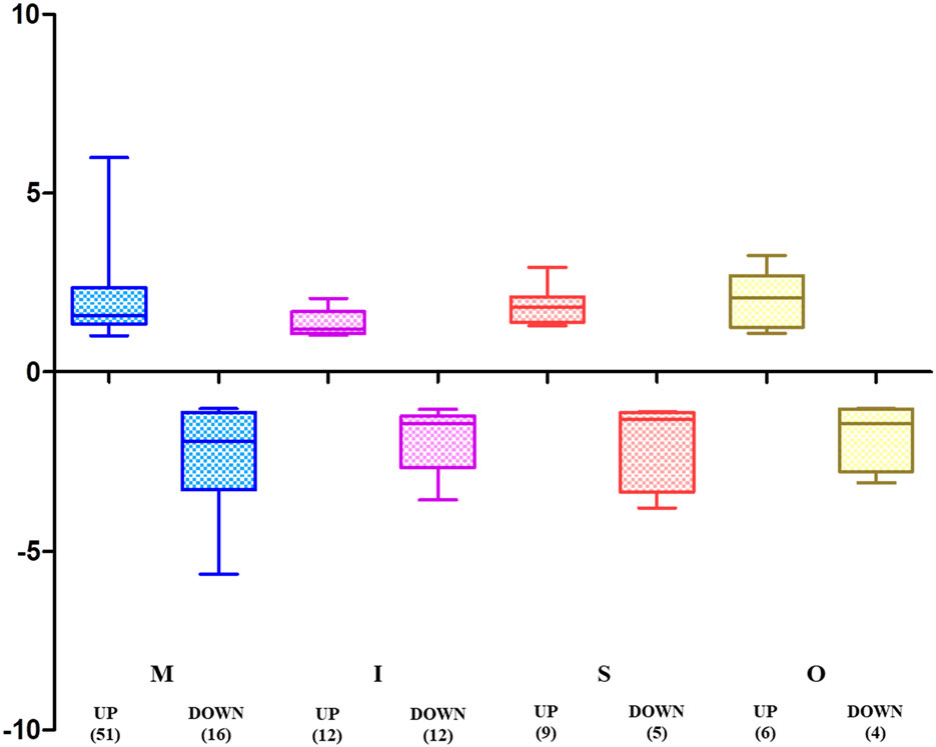
Number of tissue-specific differentially expressed lncRNAs. The abbreviations mean tissues (I, Intramuscular adipose; M, Muscles; S, Subcutaneous adipose; O, Omental adipose).

Clustering analysis using PCA analysis showed different patterns for muscle and adipose tissues. The expression of intramuscular tissue was also different from that of the other two adipose tissues (Figure 1). Hierarchical clustering analysis was performed on transcripts expression of muscle and adipose tissues in *Hanwoo* cattle. This study was similar to the muscle and adipose tissues DEG expression pattern in previous *Hanwoo* RNA-seq. Clustering analysis of the expression of transcripts showed a distinct transcript expression profile in muscle tissue expression patterns different from those of adipose tissues. Subcutaneous adipose and omental adipose tissues showed expression patterns similar, but intramuscular adipose showed an expression pattern different from those of two adipose tissues (Figure 2). We identified an expression pattern of lncRNA with muscle and adipose tissues.. The lncRNAs isolated from each tissue were 10 for omental adipose, 24 for intramuscular adipose, 14 for subcutaneous adipose, and 67 for muscle (Supplemental Tables 1 and 2). Significantly more lncRNAs were expressed in muscle tissues than in adipose tissues. We conducted PCA analysis to assess the relationship between muscle and adipose tissues. PCA analysis showed that distinguished between muscle and adipose tissues. Intramuscular adipose tissues were also distinguished from other adipose tissues (Figure 3). Expression of lncRNA differently expressed in muscle and adipose tissues were identified (Figure 4). Previous studies have examined lncRNA in several breeds of cattle. Huang et al. (2012) identified 449 putative lncRNAs using Bos taurus expressed sequence tags (ESTs) data (Huang et al. 2012). Weikard et al. (2013) identified 4,849 potential lncRNAs in the F2 of *Charolais* and *German Holstein* (Weikard et al. 2013). The expression patterns of lncRNAs were identified in skin with different pigmentation patterns. Billerey et al. (2014) identified 584 lincRNAs in the muscles of nine *Limousin* bulls (Billerey et al. 2014).

The specificity of the muscle tissues is presumed to be due to the difference from the adipose tissues. Energy is consumed mainly in muscle, and adipose tissue is the main energy-storage organization (Joe et al. 2009). The characteristics of muscle fibers affect meat quality characteristics, such as color, moisture retention, marbling, and texture (Totland et al. 1988). Adipose tissue is an important characteristic of meat and nutrition in livestock and affects animal productivity (Basu et al. 2009; Hausman et al. 2009). The amount and distribution of fatty acids is an important factor affecting meat quality in the beef industry (Brooks et al. 2011). Adipose tissue functions as an active metabolism and endocrine organ, and these are different functions that depend on the location (Kirkland and Dax 1984; Arner 1997; Kirkland et al. 1997).

Recent studies have shown that lncRNA plays an important role in the growth and differentiation of skeletal muscle. Lnc-MD1 is the lncRNA that plays an important role in myogenesis. It is specifically expressed during myoblast differentiation and leads to a transition from early- to late-stage muscle differentiation (Cesana et al. 2011). Many lncRNAs are similar to coding RNAs and are capped, spliced, and polyadenylated (Rose et al. 2011; Mercer et al. 2013). LncRNA evolved more rapidly than protein-coding genes and did not exhibit strict species conservation similar to protein-coding RNAs (Pang et al. 2006; Ulitsky et al. 2011).

### lncRNA-related bovine economic traits

**Figure 5.**
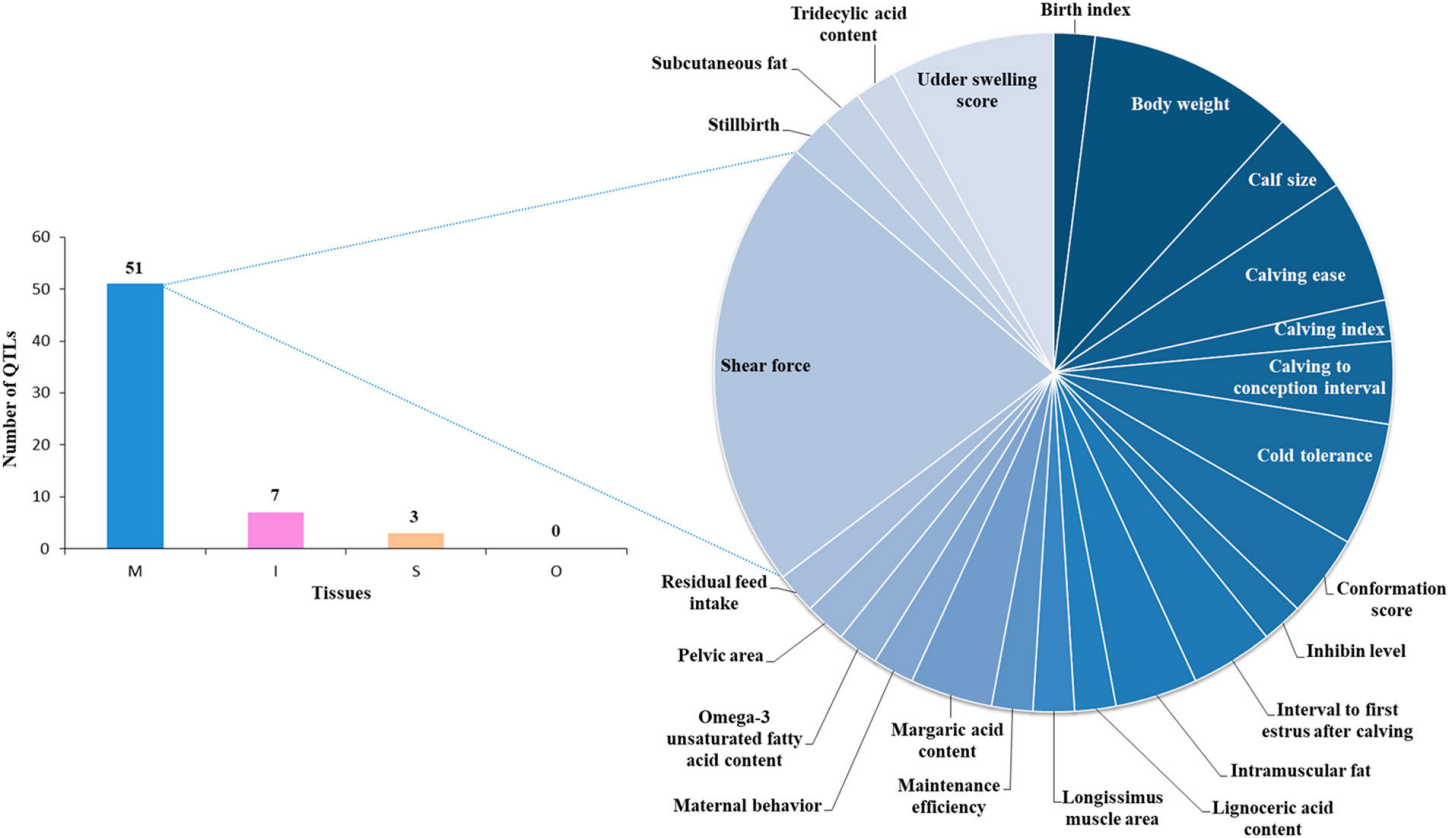
Number of QTLs associated with Hanwoo cattle economic traits in muscle and adipose tissues. The abbreviations mean tissues (I, Intramuscular adipose; M, Muscles; S, Subcutaneous adipose; O, Omental adipose).

Identification of genomic loci that glow up the complex traits was facilitated by the development of quantitative trait locus (QTL) mapping (Sonah et al. 2014). QTL is important for analyzing the putative functions of genes. Integrating QTL with gene expression or location information may enable identification of candidate genes involved in the development of a specific phenotype in cattle. 507 lincRNAs within 550 QTLs relating to meat quality and muscle related traits (Billerey et al. 2014). The Korean government introduced the beef classification standard in 1992 to assess the quality of beef (National Federation of Animal Cooperatives (NLCF) 1998). The meat-quality grading system in Korea is mainly based on the marbling score (Moon et al. 2003; Park et al. 2002). *Hanwoo* cattle have been bred and selected to improve meat quality by emphasis on greater marbling to improve taste (Han et al. 2009; Choi et al. 2013). In this study, we conducted lncRNA and QTL analysis in muscle and adipose tissues of *Hanwoo* cattle. The QTL loci associated with the meat quality and productivity of the cattle were selected by referring to the Cattle QTL Database. LncRNA extracted from muscle and intramuscular adipose overlaps with the QTL domain in terms of meat quality and muscle development characteristics. We identified 61 lncRNA overlaps with QTL, and 51 of them were in the muscle. Seven lncRNAs were identified in the intramuscular adiposes, and three lncRNAs were identified in the subcutaneous adiposes, mainly in the Shear force QTL, body weight (yearling) QTL, and udder-swelling score QTL. These results suggest that more lncRNAs were extracted from muscles than from adipose tissues (Figure 5, Table 1). The lncRNAs associated with the meat quality and productivity of *Hanwoo* cattle were identified. These results are expected to be based on various studies of *Hanwoo* cattle.

**Table 1.** List of main QTLs associated with *Hanwoo* cattle economic traits.

QTL	ID	Chr	QTL Peak	Reference	Transcript ID
Muscle					
Body weight (weaning)	24711	3	28.72	Mahdi Saatchi	ENSBTAT00000033843
	24749	3	20.34		ENSBTAT00000033843
Body weight (yearling)	22770	1	109.62	Peters SO	ENSBTAT00000009029
	24790	6	25.08	Mahdi Saatchi	ENSBTAT00000034217
					ENSBTAT00000065813
Shear force	20762	5	115.33	McClure MC	ENSBTAT00000056426
	20764	6	71.21		ENSBTAT00000034217
					ENSBTAT00000039582
					ENSBTAT00000045907
					ENSBTAT00000066034
	20770	8	27.55		ENSBTAT00000017165
	20773	8	119		ENSBTAT00000015430
	20817	25	2.61		ENSBTAT00000049975
	20824	26	40.66		ENSBTAT00000054173
	20826	27	23.61		ENSBTAT00000007942
	20833	29	56.05		ENSBTAT00000044622
Intramuscular adipose					
Shear force	20770	8	27.55	McClure MC	ENSBTAT00000017165
Subcutaneous adipose					
Body weight (birth)	24555	21	25.52	Mahdi Saatchi	ENSBTAT00000063594
Body weight (yearling)	22770	1	109.62	Peters SO	ENSBTAT00000045699

### Analysis of lncRNA-related genes

A pseudogene is a gene that has developed from genes that encode proteins. However, pseudogenes have lost the ability to produce proteins and have long been regarded as nonfunctional genomes of evolution (Poliseno 2012). The pseudogene transcript is a subclass of lncRNA. It exhibits tissue specificity and is involved in various biological processes. Previous studies have shown that lncRNA can regulate the expression of nearby genes (Rinn et al. 2007; Mercer et al. 2009).

Some of the lncRNAs differentially expressed in the muscle and adipose tissues were confirmed to be pseudogenes of overlapped genes. In addition, we classified the transcription direction of the lncRNA and the overlapped gene (Table 2). Mutation in the Coenzyme Q2 (*COQ2*) gene is associated with Primary Coenzyme Q_10_ Deficiency. It causes defects of bioenergetics and myopathy with central nervous-system involvement (Quinzii et al. 2006; López-Martín et al. 2007). The regulation of the transcriptional coactivator megakaryoblastic leukemia 1 (MKL1) by actin cytoskeleton dynamics induces mouse adipocyte differentiation mediated by the peroxisome proliferator-activated receptor γ (PPARγ), a transcriptional regulator of adipogenesis (Nobusue et al. 2014).

**Table 2.** LncRNAs information with overlapped genes.

LncRNA transcript ID	Gene ID	Gene Symbol	Loci	LncRNA strand	Gene strand
ENSBTAT00000033843	ENSBTAG00000017566	TUFT1	3:19,238,265-19,289,315	Forward	Reverse
ENSBTAT00000026486	ENSBTAG00000012307	DTNA	24:22,445,691-22,767,026	Forward	Reverse
ENSBTAT00000064565	ENSBTAG00000010542	SPIRE1	24:43,323,645-43,488,851	Forward	Reverse
ENSBTAT00000045699	ENSBTAG00000010394	MCF2L2	1:84,324,970-84,525,526	Reverse	Forward
ENSBTAT00000047753	ENSBTAG00000000687	POC1B	5:19,357,951-19,462,573	Reverse	Reverse
ENSBTAT00000065010	ENSBTAG00000002630	MKL1	5:112,261,806-112,372,282	Reverse	Reverse
ENSBTAT00000066034	ENSBTAG00000005744	COQ2	6:99,812,238-99,839,387	Reverse	Reverse
ENSBTAT00000015430	ENSBTAG00000046602	PALM2	8:101,018,440-101,182,488	Reverse	Forward
ENSBTAT00000065436	ENSBTAG00000009427	PPM1D	19:12,602,357-12,662,178	Reverse	Reverse
ENSBTAT00000049295	ENSBTAG00000024801	RANBP17	20:2,680,574-3,054,892	Reverse	Forward
ENSBTAT00000065849	ENSBTAG00000035705	MTMR8	X:101,228,033-101,491,280	Reverse	Forward

We also classified lncRNAs that are opposite to the transcription direction of the overlapped genes. The pairing of sense and antisense transcription leads to the formation of double-stranded RNA (dsRNA), which can trigger activation of the RNA interference (RNAi) pathway. The protein component of the RNAi pathway, Dicer, splits the dsRNA into smaller pieces known as small interfering RNAs (siRNAs). This siRNA is integrated into the RNA-induced silencing complex (RISC), which degrades and inhibits the mRNA of the parent coding gene (Tam et al. 2008; Watanabe et al. 2008). Two lncRNAs were identified as pseudogenes of muscle-associated genes. β-karyopherin genes include the RAN Binding Protein 17 (*RANBP17*) gene. In eukaryotes, *β* karyopherin protein mediates the nuclear cytoplasmic transport of macromolecules. In addition, Mouse Ranbp17 mRNA has high expression in skeletal muscle (Quan et al. 2008). Dystrobrevin-α, encoded by Dtna, belongs to a family of dystrophin-related proteins. The Dystrobrevin Alpha (*DTNA*) gene is highly expressed in skeletal muscle and is related to muscle diseases (Rees et al. 2007). Therefore, the lncRNAs identified here may have a crucial role for expression of genes at the specific locus that can functionally affect skeletal muscle, which could help clarify the function of the corresponding lncRNA.

### Conclusion

This study is the first paper to profile tissue-specific lncRNAs by using comparative analysis of muscle and adipose tissue in *Hanwoo* cattle. We compared the bovine lncRNA found in the previous study using the transcript data of *Hanwoo* cattle and identified 76 lncRNAs. Expression patterns of lncRNAs in each tissue were identified. Many lncRNAs were identified in muscles, which have biological metabolism characteristics different from those of adipose tissue. We found the lncRNAs located in the QTL locus. QTL loci related to the meat quality and productivity of the cattle were selected for analysis. These lncRNAs were associated with cattle economic characteristics, such as shear force and body weight. In addition, we found the pseudogenes that are predicted to affect function in the muscles of *Hanwoo* cattle. In conclusion, we conducted a basic study on the characteristics of lncRNA that is expressed specifically in the skeletal muscle and adipose tissues of *Hanwoo* cattle. We also identified the candidate lncRNA for the economic traits of *Hanwoo* cattle. This study will contribute to metabolic function studies by cattle organizations.

## Supplementary Material

Supplemental_file.docx
