# Supplementary material for: Comparison of long noncoding RNA between muscles and adipose tissues in Hanwoo beef cattle
Source: Anim Cells Syst (Seoul). 2018 Dec 20;23(1):50–8. doi: 10.1080/19768354.2018.1512522 (PMC6394308; doi:10.1080/19768354.2018.1512522)
Supplement: Supplemental_file.docx [file TACS_A_1512522_SM1937.docx]

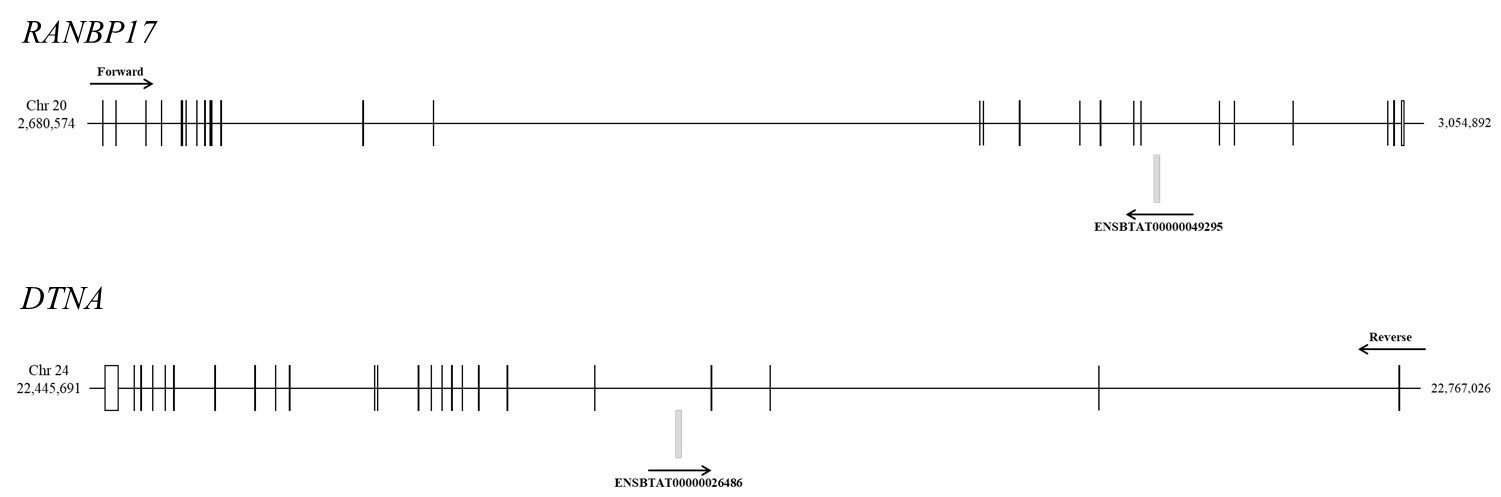


Supplement figure1. Muscle tissue-specific lncRNAs nearby to muscle-associated genes

Supplement Table1. List of differentially expressed lncRNA of Hanwoo cattle in muscle.

| Transcript ID | Loci | log2FC | p-value | nearby gene | Transcript ID | Loci | log2FC | p-value | nearby gene |
| --- | --- | --- | --- | --- | --- | --- | --- | --- | --- |
| Muscle | | | | | | | | | |
| ENSBTAT00000045699 | 1:84324177-84325181 | 2.26 | 0.00E+00 | MCF2L2 | ENSBTAT00000021450 | 12:33647815-33648405 | 1.73 | 1.49E-08 |  |
| ENSBTAT00000009029 | 1:80940147-80940854 | 2.03 | 3.68E-11 |  | ENSBTAT00000034807 | 12:18061076-18062037 | 1.53 | 4.92E-07 |  |
| ENSBTAT00000008958 | 1:77092288-77092759 | 1.64 | 8.54E-04 |  | ENSBTAT00000027547 | 13:47904560-47905533 | 1.33 | 5.31E-20 |  |
| ENSBTAT00000018339 | 2:107981292-107982291 | -3.50 | 0.00E+00 |  | ENSBTAT00000050065 | 13:43280032-43281472 | 1.49 | 5.81E-07 |  |
| ENSBTAT00000013153 | 2:84413662-84414878 | -2.08 | 1.20E-80 |  | ENSBTAT00000039303 | 13:61234714-61235657 | 3.50 | 8.44E-06 |  |
| ENSBTAT00000007335 | 2:119050744-119051409 | 1.56 | 1.67E-30 |  | ENSBTAT00000064621 | 14:20738814-20740407 | 1.20 | 1.74E-116 |  |
| ENSBTAT00000045444 | 2:122343234-122344583 | 1.25 | 3.11E-11 |  | ENSBTAT00000064839 | 15:74915626-74916613 | 3.16 | 4.94E-219 |  |
| ENSBTAT00000024323 | 2:5015011-5016211 | 1.27 | 7.30E-08 |  | ENSBTAT00000055155 | 15:51667478-51669427 | -1.14 | 2.20E-21 |  |
| ENSBTAT00000033843 | 3:19259499-19260488 | 1.55 | 3.00E-80 | TUFT1 | ENSBTAT00000039377 | 16:64843773-64844924 | 1.23 | 1.12E-45 |  |
| ENSBTAT00000056069 | 3:111030118-111030977 | 1.01 | 5.94E-06 |  | ENSBTAT00000027978 | 17:28834268-28835511 | 1.77 | 1.76E-22 |  |
| ENSBTAT00000025667 | 4:33507617-33508901 | 4.28 | 8.00E-04 |  | ENSBTAT00000006571 | 18:43927222-43929798 | 5.99 | 0.00E+00 |  |
| ENSBTAT00000063416 | 5:32529439-32529956 | 2.13 | 4.57E-51 |  | ENSBTAT00000065436 | 19:12622717-12623538 | 1.14 | 4.39E-45 | PPM1D |
| ENSBTAT00000004154 | 5:102100648-102101142 | 1.03 | 4.98E-37 |  | ENSBTAT00000049295 | 20:2985749-2986486 | 2.35 | 1.62E-189 | RANBP17 |
| ENSBTAT00000056426 | 5:100719638-100720427 | 3.40 | 7.61E-09 |  | ENSBTAT00000047705 | 20:55967804-55968473 | 2.94 | 2.33E-36 |  |
| ENSBTAT00000047753 | 5:19459449-19461191 | 2.36 | 8.24E-07 | POC1B | ENSBTAT00000064456 | 20:21814396-21815526 | -5.65 | 2.19E-04 |  |
| ENSBTAT00000045907 | 6:85640347-85641522 | 1.52 | 5.95E-84 |  | ENSBTAT00000065287 | 21:19391574-19391762 | 1.74 | 2.63E-111 |  |
| ENSBTAT00000065813 | 6:35008595-35010340 | -1.10 | 1.55E-32 |  | ENSBTAT00000045888 | 22:48146336-48146712 | -1.10 | 9.28E-60 |  |
| ENSBTAT00000034217 | 6:27328572-27329745 | 1.10 | 1.51E-27 |  | ENSBTAT00000052413 | 22:49660685-49661474 | 2.07 | 1.59E-05 |  |
| ENSBTAT00000039582 | 6:90636202-90637197 | -2.24 | 2.24E-25 |  | ENSBTAT00000055663 | 23:27614103-27615194 | -1.64 | 1.93E-133 |  |
| ENSBTAT00000066034 | 6:99833916-99835304 | 1.39 | 4.83E-10 | COQ2 | ENSBTAT00000039090 | 23:29411406-29412041 | 1.24 | 7.37E-24 |  |
| ENSBTAT00000013213 | 7:39875477-39876428 | 2.48 | 6.83E-115 |  | ENSBTAT00000064565 | 24:43454302-43455153 | 1.55 | 4.17E-12 | SPIRE1 |
| ENSBTAT00000011804 | 7:22831359-22831913 | 1.34 | 3.29E-42 |  | ENSBTAT00000026486 | 24:22586221-22586611 | -4.90 | 8.61E-04 | DTNA |
| ENSBTAT00000052414 | 7:45556210-45557580 | 1.35 | 4.78E-14 |  | ENSBTAT00000049975 | 25:1167351-1168312 | 1.03 | 1.27E-15 |  |
| ENSBTAT00000017165 | 8:31871390-31872132 | 1.98 | 1.22E-95 |  | ENSBTAT00000010367 | 26:11110265-11111041 | 1.84 | 1.59E-11 |  |
| ENSBTAT00000015430 | 8:101049650-101050143 | -1.97 | 6.71E-05 | PALM2 | ENSBTAT00000054173 | 26:31870225-31871586 | 2.84 | 4.93E-09 |  |
| ENSBTAT00000049099 | 9:18473883-18475244 | -1.86 | 8.26E-141 |  | ENSBTAT00000007942 | 27:15623109-15623317 | 1.05 | 1.86E-40 |  |
| ENSBTAT00000055377 | 9:85002458-85003612 | 1.54 | 5.20E-13 |  | ENSBTAT00000016095 | 28:3187636-3188295 | 2.76 | 1.47E-149 |  |
| ENSBTAT00000010270 | 10:76992202-76994112 | 2.61 | 1.52E-210 |  | ENSBTAT00000063057 | 29:272264-273010 | -2.60 | 0.00E+00 |  |
| ENSBTAT00000003768 | 10:99351222-99352544 | 1.58 | 2.59E-24 |  | ENSBTAT00000044622 | 29:40214778-40215227 | -1.01 | 4.32E-153 |  |
| ENSBTAT00000025677 | 11:84076854-84078206 | -1.09 | 4.04E-62 |  | ENSBTAT00000063679 | 29:31113681-31114595 | 1.34 | 2.76E-05 |  |
| ENSBTAT00000064166 | 11:72059857-72060691 | -1.44 | 3.17E-06 |  | ENSBTAT00000022189 | X:66722399-66723397 | -3.85 | 0.00E+00 |  |
| ENSBTAT00000007745 | 11:47210063-47210732 | 2.38 | 7.16E-05 |  | ENSBTAT00000028000 | X:86605969-86606883 | 2.04 | 2.80E-43 |  |
| ENSBTAT00000039063 | 11:93927186-93927978 | 1.35 | 7.48E-04 |  | ENSBTAT00000017703 | X:94378601-94380496 | 1.53 | 2.03E-08 |  |
| ENSBTAT00000064261 | 12:15274614-15276029 | 2.52 | 1.84E-44 |  |  |  |  |  |  |

Supplement Table2. List of differentially expressed lncRNA of Hanwoo cattle in tissues.

| Transcript ID | Loci | log2FC | p-value | Nearby gene | Transcript ID | Loci | log2FC | p-value | Nearby gene |
| --- | --- | --- | --- | --- | --- | --- | --- | --- | --- |
| Intramuscular adipose | | | | | Subcutaneous adipose | | | | |
| ENSBTAT00000045699 | 1:84324177-84325181 | 1.06 | 1.69E-225 | MCF2L2 | ENSBTAT00000045699 | 1:84324177-84325181 | -1.10 | 0.00E+00 | MCF2L2 |
| ENSBTAT00000008958 | 1:77092288-77092759 | -1.58 | 3.53E-05 |  | ENSBTAT00000018339 | 2:107981292-107982291 | 1.92 | 1.64E-127 |  |
| ENSBTAT00000018339 | 2:107981292-107982291 | 1.75 | 5.21E-115 |  | ENSBTAT00000027478 | 4:58724265-58724900 | 1.54 | 2.45E-50 |  |
| ENSBTAT00000040333 | 2:112675322-112675995 | 1.10 | 1.94E-93 |  | ENSBTAT00000039582 | 6:90636202-90637197 | 1.30 | 2.47E-06 |  |
| ENSBTAT00000013153 | 2:84413662-84414878 | 1.05 | 7.83E-15 |  | ENSBTAT00000013033 | 9:29703282-29703781 | -2.90 | 1.07E-04 |  |
| ENSBTAT00000027478 | 4:58724265-58724900 | -1.54 | 1.82E-76 |  | ENSBTAT00000010270 | 10:76992202-76994112 | -1.14 | 1.01E-94 |  |
| ENSBTAT00000065010 | 5:112286611-112287081 | -3.02 | 8.00E-04 | MKL1 | ENSBTAT00000007745 | 11:47210063-47210732 | 1.93 | 3.67E-04 |  |
| ENSBTAT00000065813 | 6:35008595-35010340 | 1.02 | 3.83E-20 |  | ENSBTAT00000064839 | 15:74915626-74916613 | 1.80 | 2.56E-116 |  |
| ENSBTAT00000017165 | 8:31871390-31872132 | -1.13 | 5.16E-58 |  | ENSBTAT00000006571 | 18:43927222-43929798 | -1.32 | 2.24E-178 |  |
| ENSBTAT00000049099 | 9:18473883-18475244 | 1.48 | 1.52E-54 |  | ENSBTAT00000065287 | 21:19391574-19391762 | 1.44 | 1.95E-88 |  |
| ENSBTAT00000007745 | 11:47210063-47210732 | -3.56 | 1.11E-13 |  | ENSBTAT00000063594 | 21:20144494-20144827 | -3.79 | 1.75E-04 |  |
| ENSBTAT00000064166 | 11:72059857-72060691 | 1.94 | 3.03E-06 |  | ENSBTAT00000063057 | 29:272264-273010 | 1.35 | 8.79E-134 |  |
| ENSBTAT00000039063 | 11:93927186-93927978 | -1.29 | 6.64E-05 |  | ENSBTAT00000022189 | X:66722399-66723397 | 2.29 | 0.00E+00 |  |
| ENSBTAT00000034807 | 12:18061076-18062037 | -1.20 | 8.03E-07 |  | ENSBTAT00000024088 | X:24737905-24739261 | 2.93 | 7.59E-08 |  |
| ENSBTAT00000064621 | 14:20738814-20740407 | -1.35 | 2.20E-210 |  | Omental adipose | | | | |
| ENSBTAT00000064839 | 15:74915626-74916613 | -3.31 | 0.00E+00 |  | ENSBTAT00000018339 | 2:107981292-107982291 | 2.20 | 5.72E-152 |  |
| ENSBTAT00000006571 | 18:43927222-43929798 | 1.20 | 1.03E-101 |  | ENSBTAT00000039582 | 6:90636202-90637197 | 1.08 | 5.54E-05 |  |
| ENSBTAT00000065287 | 21:19391574-19391762 | -1.27 | 3.14E-99 |  | ENSBTAT00000007745 | 11:47210063-47210732 | 3.26 | 1.13E-06 |  |
| ENSBTAT00000055663 | 23:27614103-27615194 | 1.19 | 6.54E-47 |  | ENSBTAT00000064839 | 15:74915626-74916613 | 1.93 | 3.79E-127 |  |
| ENSBTAT00000054173 | 26:31870225-31871586 | -1.51 | 1.76E-06 |  | ENSBTAT00000006571 | 18:43927222-43929798 | -1.00 | 1.48E-103 |  |
| ENSBTAT00000063057 | 29:272264-273010 | 1.30 | 1.68E-129 |  | ENSBTAT00000047705 | 20:55967804-55968473 | -1.82 | 9.14E-37 |  |
| ENSBTAT00000022189 | X:66722399-66723397 | 2.06 | 0.00E+00 |  | ENSBTAT00000065672 | 23:34219298-34219825 | -1.06 | 1.50E-268 |  |
| ENSBTAT00000040092 | X:79506820-79508013 | -1.03 | 2.18E-44 |  | ENSBTAT00000063057 | 29:272264-273010 | 1.30 | 2.33E-125 |  |
| ENSBTAT00000065849 | X:101428040-101428600 | 1.11 | 1.33E-28 | MTMR8 | ENSBTAT00000022189 | X:66722399-66723397 | 2.50 | 0.00E+00 |  |
|  |  |  |  |  | ENSBTAT00000024088 | X:24737905-24739261 | -3.08 | 7.22E-18 |  |

Supplement Table3. List of QTLs associated with Hanwoo cattle economic traits in muscle.

| **QTL** | **ID** | **Chr** | **QTL Peak** | **Reference** | **Transcript ID** |
| --- | --- | --- | --- | --- | --- |
| **Muscle** | | | | | |
| Birth index | 30537 | 18 | 46.8 | Höglund JK | ENSBTAT00000006571 |
| Body weight (weaning) | 24711 | 3 | 28.72 | Mahdi Saatchi | ENSBTAT00000033843 |
|  | 24749 | 3 | 20.34 |  | ENSBTAT00000033843 |
|  |  |  |  |  | ENSBTAT00000009029 |
|  | 24790 | 6 | 25.08 |  | ENSBTAT00000034217 |
|  |  |  |  |  | ENSBTAT00000065813 |
| Calf size | 15211 | 20 | 26.17 | Sahana G | ENSBTAT00000064456 |
|  | 15212 |  |  |  | ENSBTAT00000064456 |
| Calving ease | 106440 | 10 | 109.97 | Michenet A | ENSBTAT00000003768 |
|  | 15213 | 20 | 26.17 | Sahana G | ENSBTAT00000064456 |
|  | 30569 | 26 | 45.45 | Höglund JK | ENSBTAT00000054173 |
| Calving index | 15210 | 20 | 26.17 | Sahana G | ENSBTAT00000064456 |
| Calving to conception interval | 126853 | 6 | N/A | Müller MP | ENSBTAT00000039582 |
|  | 126888 | 23 | N/A |  | ENSBTAT00000039090 |
| Cold tolerance | 31181 | 7 | 61.47 | Howard JT | ENSBTAT00000052414 |
|  |  |  |  |  | ENSBTAT00000011804 |
|  |  |  |  |  | ENSBTAT00000013213 |
| Conformation score | 102040 | 23 | N/A | Mao X | ENSBTAT00000039090 |
|  |  |  |  |  | ENSBTAT00000055663 |
| Inhibin level | 30700 | 26 | 45.45 | Fortes MRS | ENSBTAT00000054173 |
| Interval to first estrus after calving | 126854 | 6 | N/A | Müller MP | ENSBTAT00000039582 |
|  | 126889 | 23 | N/A |  | ENSBTAT00000039090 |
| Intramuscular fat | 22866 | 5 | 40.42 | Peters SO | ENSBTAT00000063416 |
|  | 37965 | 18 | 57.55 | Barendse W | ENSBTAT00000006571 |
| Lignoceric acid content | 19707 | 7 | 55.04 | Saatchi M | ENSBTAT00000052414 |
| Longissimus muscle area | 126448 | 8 | N/A | de Oliveira Silva RM | ENSBTAT00000017165 |
| Maintenance efficiency | 35978 | 9 | 92.28 | de Oliveira PS | ENSBTAT00000055377 |
| Margaric acid content | 19759 | 29 | 46.64 | Saatchi M | ENSBTAT00000063679 |
|  |  |  |  |  | ENSBTAT00000044622 |
| Maternal behavior | 106675 | 26 | 46.13 | Michenet A | ENSBTAT00000054173 |
| Omega-3 unsaturated fatty acid content | 19735 | 7 | 55.04 | Saatchi M | ENSBTAT00000052414 |
| Pelvic area | 106481 | 26 | 43.84 | Michenet A | ENSBTAT00000054173 |
|  | 56461 | 14 | 31.48 | Saatchi M | ENSBTAT00000064621 |
| Shear force | 20762 | 5 | 115.33 | McClure MC | ENSBTAT00000056426 |
|  | 20764 | 6 | 71.21 | McClure MC | ENSBTAT00000034217 |
|  |  |  |  |  | ENSBTAT00000039582 |
|  |  |  |  |  | ENSBTAT00000045907 |
|  |  |  |  |  | ENSBTAT00000066034 |
|  | 20770 | 8 | 27.55 |  | ENSBTAT00000017165 |
|  | 20773 | 8 | 119 |  | ENSBTAT00000015430 |
|  | 20817 | 25 | 2.61 |  | ENSBTAT00000049975 |
|  | 20824 | 26 | 40.66 |  | ENSBTAT00000054173 |
|  | 20826 | 27 | 23.61 |  | ENSBTAT00000007942 |
|  | 20833 | 29 | 56.05 |  | ENSBTAT00000044622 |
| Stillbirth | 30521 | 12 | 40.36 | Höglund JK | ENSBTAT00000021450 |
| Subcutaneous fat | 20703 | 14 | 5.62 | Veneroni-Gouveia G | ENSBTAT00000064621 |
| Tridecylic acid content | 19626 | 15 | 41.81 | Saatchi M | ENSBTAT00000055155 |
| Udder swelling score | 106727 | 8 | 111.09 | Michenet A | ENSBTAT00000015430 |
|  | 106730 | 10 | 110.77 |  | ENSBTAT00000003768 |
|  | 106757 | 29 | 42.89 |  | ENSBTAT00000063679 |
|  | 106760 | 29 | 54.42 |  | ENSBTAT00000044622 |

Supplement Table4. List of QTLs associated with Hanwoo cattle economic traits in Intramuscular adipose

| QTL | ID | Chr | QTL Peak | Reference | Transcript ID |
| --- | --- | --- | --- | --- | --- |
| **Intramuscular adipose** | | | | | |
| Average daily gain | 22798 | 15 | 97.09 | Peters SO | ENSBTAT00000064839 |
| Longissimus muscle area | 126448 | 8 | N/A | de Oliveira Silva RM | ENSBTAT00000017165 |
|  | 126451 | 15 | N/A |  | ENSBTAT00000064839 |
| Residual feed intake | 35233 | 11 | 112.06 | de Oliveira PS | ENSBTAT00000039063 |
|  | 56461 | 14 | 31.48 | Saatchi M | ENSBTAT00000064621 |
| Shear force | 20770 | 8 | 27.55 | McClure MC | ENSBTAT00000017165 |
| Subcutaneous fat | 20703 | 14 | 5.62 | Veneroni-Gouveia G | ENSBTAT00000064621 |

.

Supplement Table5. List of QTLs associated with Hanwoo cattle economic traits in Subcutaneous adipose

| QTL | ID | Chr | QTL Peak | Reference | Transcript ID |
| --- | --- | --- | --- | --- | --- |
| **Subcutaneous adipose** | | | | | |
| Body weight (birth) | 24555 | 21 | 25.52 | Mahdi Saatchi | ENSBTAT00000063594 |
| Body weight (yearling) | 22770 | 1 | 109.62 | Peters SO | ENSBTAT00000045699 |
| Trans-6/9-C18:1 fatty acid content | 20504 | 1 | 80.99 | Saatchi M | ENSBTAT00000045699 |
